# Intra Vaginal Respiration Recommended in Some Cases of Parturition Where the Child’s Life Is in Danger from Pressure on the Cord

**Published:** 1849-07

**Authors:** 


ART. II.—*Intra Vaginal respiration recommended in some cases of Parturition where the child's life is in danger from pressure on the cord.* By M. M'Culloch, M. D., Lecturer on Midwifery and Diseases of Women and Children, University of M'Gill College, Montreal, Canada.

I beg, through the medium of the *British American Journal*, to make known a new mode of practice by which the amount of infant mortality will be lessened in many cases in which death from pressure on the cord is otherwise inevitable. If we take the average of French, German, and British practice, we find by the most authentic statistics that danger to the life of the child from this cause occurs in breech presentations once in 53 labors; in presentations of the superior extremeties, after turning, once in 261, and where the cord presents once in 245 cases. Thus we have in 649 labors 12 presentations of the breech, 7 of the inferior extremeties, 2½ of the superior extremeties, and 2½ of the cord, occasioning a loss of life at birth of about two per cent. of all children born. This great mortality I think I have succeeded in diminishing in my own practice in three footling cases within the last eight months, by enabling the child to breathe while the head was still within the cavity of the pelvis after the pulsation in the cord had been several minutes extinct. And this novel state of existence was maintained by keeping the child's mouth open with my finger, and the perineum expanded to allow air to have free access to the vagina. In future I shall endeavor to accomplish this object with greater certainty by introducing into the mouth the end of the largest sized gum elastic male catheter, with several perforations in it, and continue as before to allow as much air as possible to approach the face and nostrils. In all cases of malposition in which the practice I have suggested becomes desirable, the chance of respiration being established will be greater if the means are used before the circulation becomes feeble in the umbilical vessels; and if the child is born alive in the absence of pulsation in them, it may be fairly conceded that we have been successful in our efforts to preserve life. Some cases may occur where a small sized male catheter with a suitable curve, might be used with advantage as a tracheal pipe. I need scarcely add that the infant's body should be kept warm, gentle traction employed, and all the other ordinary means of exciting contraction of the uterus used until the delivery is completed.

If the practice I have here recommended is found to answer in other hands as it has in my own, something will have been achieved in obsteteric science, in circumstances where the most skilful have hitherto frequently had to lament their inability to save the child's life.

Montreal, April 20, 1849.

